# Extract of Fructus Cannabis Ameliorates Learning and Memory Impairment Induced by D-Galactose in an Aging Rats Model

**DOI:** 10.1155/2017/4757520

**Published:** 2017-10-19

**Authors:** Ning-Yuan Chen, Cheng-Wu Liu, Wei Lin, Yi Ding, Zhang-ya Bian, Ling Huang, Hao Huang, Kai-Hui Yu, Si-Bang Chen, Yu Sun, Lei Wei, Jun-Hua Peng, Shang-Ling Pan

**Affiliations:** ^1^Department of Pathophysiology, School of Preclinical Medicine, Guangxi Medical University, Nanning, China; ^2^Guangxi Colleges and Universities Key Laboratory of Human Development and Disease Research, 22 Shuangyong Road, Nanning, Guangxi 530021, China; ^3^Department of Neurological Rehabilitation, Guangxi Jiangbin Hospital, Nanning, Guangxi 530021, China; ^4^The First Clinical Medical School, Guangxi Medical University, Nanning, Guangxi 530021, China

## Abstract

Hempseed (*Cannabis sativa* L.) has been used as a health food and folk medicine in China for centuries. In the present study, we sought to define the underlying mechanism by which the extract of Fructus Cannabis (EFC) protects against memory impairment induced by D-galactose in rats. To accelerate aging and induce memory impairment in rats, D-galactose (400 mg/kg) was injected intraperitoneally once daily for 14 weeks. EFC (200 and 400 mg/kg) was simultaneously administered intragastrically once daily in an attempt to slow the aging process. We found that EFC significantly increased the activity of superoxide dismutase, while lowering levels of malondialdehyde in the hippocampus. Moreover, EFC dramatically elevated the organ indices of some organs, including the heart, the liver, the thymus, and the spleen. In addition, EFC improved the behavioral performance of rats treated with D-galactose in the Morris water maze. Furthermore, EFC inhibited the activation of astrocytes and remarkably attenuated phosphorylated tau and suppressed the expression of presenilin 1 in the brain of D-galactose-treated rats. These findings suggested that EFC exhibits beneficial effects on the cognition of aging rats probably by enhancing antioxidant capacity and anti-neuroinflammation, improving immune function, and modulating tau phosphorylation and presenilin expression.

## 1. Introduction

In the past decades, the population birth rates are decreasing, and human lifespan is expanding. As a result, 11% of the world's population is over the age of 60, and this proportion is expected to double by 2050 [1]. As our populations are rapidly aging, there is increasing awareness of the age-related cognitive deficits caused by diseases associated with aging, such as Alzheimer's disease (AD). New therapies are urgently needed to treat or delay these diseases.

Among aging theories, free radical theory is one of the most widely accepted explanations for senescence [2]. This theory argues that aging leads to a disturbance of oxygen free radical metabolism, and the excess oxygen free radicals attack cell membranes by peroxidating lipids and increase malondialdehyde (MDA) levels. At the same time, the activity of superoxide dismutase (SOD), a scavenger of superoxide anion, is reduced [3]. High doses of D-galactose (D-gal) result in accumulation of galactitol, osmotic stress, and reactive oxygen species (ROS) that aggravate aging [4]. Meanwhile, D-gal overload in the brain subsequently causes neurotoxicity such as oxidative stress and neuroinflammation [5, 6] and eventually leads to cognitive deficits. Hence, the administration of D-gal has been widely used for establishing animal aging model [7] and to study age-related neurodegenerative diseases [8].

Hempseed is the fructus of* Cannabis sativa*, which has been commonly used as a Chinese traditional medicine and nonstaple food for centuries. It contains a variety of essential fatty acids, such as linoleic acid (*ω*-6) and alpha-linolenic acid (*ω*-3) [9]. Thus, extract of Fructus Cannabis (EFC) may have great utility as a health food with the potential to prevent several lipid-associated diseases [10]. Hempseed also contains antioxidant peptides that reduce oxidative stress in spontaneously hypertensive rats [11]. In addition, dietary hempseed reduces platelet aggregation because it is enriched in polyunsaturated fatty acids [12]. Hempseed meal protein hydrolysate prevents hypertension and lowers blood pressure in spontaneously hypertensive rats [13]. However, whether EFC protects against the cognitive deficits associated with aging is unknown.

Residents dwelling in Hongshuihe River basin, Guangxi Province, have used hempseed as a popular health food for centuries. In previous studies, we evaluated the cognitive function of longevity population in Hongshuihe River basin by using the psychometric tests of the mini-mental state examination (MMSE) [14]. The results revealed that long-lived individuals with good cognition might be associated with the benefit of dietary hempseed. It is thus reasonable to assume that EFC may have potential neuroprotective effect against brain aging and memory impairments. In the current study, we aim to determine if EFC improves learning and memory in rats treated with D-gal and define the mechanism underlying its protective effects on brain aging.

## 2. Materials and Methods

### 2.1. Chemicals and Reagents

Hempseed was purchased in farm product market in Bama County, Guangxi Province, China. D-Galactose was purchased from Sigma-Aldrich (Hamburg, Germany). SOD and MDA kits were obtained from Nanjing Jiancheng Bioengineering Institute (Nanjing, China). Primary antibodies for total tau, phosphorylated tau, and presenilin-1 were purchased from Abcam (Cambridge, MA, US), and anti-glial fibrillary acidic protein (GFAP) antibody was from Sigma-Aldrich (St. Louis, MO, USA). Alexa Fluor 555 antibody was purchased from Thermo Fisher Scientific (Waltham, MA, USA). GAPDH was from Good Here Biotechnology (Hang Zhou, China). Goat anti-rabbit IgG and goat anti-mouse IgG were purchased from LI-COR Biosciences (Lincoln, NE, US).

### 2.2. Preparation of EFC

Hempseed (*Cannabis sativa* L.) was identified by Dr. Zhenguo Zhong, a professor of Guangxi University of Traditional Chinese Medicine. EFC was prepared as described previously [15]. Briefly, hempseed was air-dried and was dehulled using a laboratory huller machine (HT80-20, Shan Dong Instrument, China). Then the seeds were extracted with 90% ethanol for 24 hours and filtrated. The filtrate was concentrated to dry and the total yield was 5 g (1%) in terms of starting materials. EFC was dissolved in saline prior to administration at doses of 200 mg/kg and 400 mg/kg.

### 2.3. HPLC-MS Analysis of Components in EFC

The ethanol extract was subjected to column chromatography under reduced pressure on silica gel, eluted with a chloroform-methanol solvent system. Its components were analyzed by high performance liquid chromatography (HPLC) with a Lichrospher 100 RP chromatographic column and identified according to mass spectrometry (MS). The mobile phase consisted of acetonitrile and ammonium acetate (12 : 88) at a flow rate of 0.8 ml/min during a run time of 34 minutes and the column temperature was 35°C.

### 2.4. Animals and Drug Administration

Three-month-old Sprague-Dawley (SD) male rats (SPF grade, wt 259–278 g) were obtained from the Experimental Animal Center, Guangxi Medical University (SYXK 2009-0002). The rats were housed in ventilated cages at 24 ± 2°C, with a humidity of 50 ± 10%, under a 12 h light/dark cycle. Standard diet and water were available ad libitum. The experimental protocols were approved by the Institutional Ethical Committee of Guangxi Medical University (approval number 20110501202).

After 1 week of acclimatization to vivarium conditions, the animals were screened for the Morris water maze test. Rats that reached the hidden platform within 90 seconds were selected. Forty eligible rats were randomly divided into five groups with 8 rats for each: (1) control group; (2) EFC group (400 mg/kg); (3) D-gal group (D-gal-induced aging group); (4) D-gal + EFC (200 mg/kg) group; and (5) D-gal + EFC (400 mg/kg) group. To establish the animal model of aging, rats in the D-gal group were injected intraperitoneally with D-gal at a dose of 400 mg/kg/day for 14 weeks, and the rats in control and EFC (400 mg/kg) groups were injected with an equal volume of normal saline. At the same time, EFC was administered intragastrically once per day for 14 weeks.

### 2.5. Morris Water Maze Test

After 14 weeks of drug administration, rats (*n* = 8 each group) were tested in Morris water maze as described previously [16]. The experimental apparatus consisted of a circular pool filled with water (24 ± 2°C) which was rendered opaque by adding ink. The hidden platform was submerged below the water surface in the center of target quadrant. During 5 days of navigation training, the rats underwent 4 trials per day with four quadrants of rotational starting points and allowed a maximum of 90 s to search for the hidden platform (escape latency). On the sixth day, the probe trial was performed by removing the platform and allowing each rat to swim freely for 60 s. The escape latency and the number of platform crossings as well as time spent in the target quadrant were recorded by a video camera mounted on the ceiling, and data were analyzed by using SMART Behavior Analysis System.

### 2.6. Observation of the General Appearance, Body Weight, and Organ Index

The general appearance of the rats was observed daily throughout the experiment, and body weight was recorded weekly. Rats were sacrificed after behavioral tests and brain tissues were collected for biochemical analysis or western blotting analysis. Hippocampus tissues were immediately collected and assayed as described below. The hearts and livers, the spleens, and the thymus glands were gathered and weighed. The organ index was calculated using the following equation: organ index = organ weight (g)/body weight (g) × 100%.

### 2.7. SOD Activity and MDA Level Measurements in the Hippocampus

Hippocampus tissues were prepared in ice-cold physiological saline and centrifuged at 1.2 × 10^4^ rpm for 15 minutes at 4°C, and supernatants were collected for analysis. SOD activities and MDA levels in the hippocampus were analyzed according to the manufacturer's instructions.

### 2.8. Immunofluorescent Staining

It is well known that the activation of astrocytes is indicated as increased glial fibrillary acidic protein (GFAP) level. The protocol for immunofluorescent staining has been described previously [17]. Sections were incubated at 4°C overnight with a mouse monoclonal anti-GFAP antibody (1 : 400, Sigma-Aldrich, MO, USA). Following incubation with primary antibody, the sections were rinsed in TBS and then incubated with goat anti-mouse IgG Alexa Fluor 555 antibody (1 : 1000, Waltham, MA, USA) for 1 h at room temperature. Images from the CA1 region of hippocampus in each section were observed with a microscope (ECLIPSE Ti-s, Nikon, Japan) and digital sight camera (ECLIPSE TS100, Nicon, Japan). The staining of GFAP-positive cells was measured using computerized planimetry (Image-Pro Plus Media Cybernetics, Inc., Rockville, USA). Results of 4 separate measurements for each section were expressed as mean ± SE; statistical differences were assessed by one-way ANOVA tests.

### 2.9. Western Blot Analysis

The proteins of hippocampus tissue were separated by SDS-PAGE and transferred to polyvinylidene fluoride (PVDF) membranes. The membranes were incubated overnight at 4°C with the primary antibodies appropriately diluted. The secondary antibody was diluted 1 : 3000 in Tris-buffered saline with Tween 20 (TBST). LI-COR Odyssey platform (infrared laser) was used to detect the immune-reactive bands. GAPDH was used as an internal control, and the intensity of the bands was quantified using Odyssey V3.0 software.

### 2.10. Statistical Analysis

Results were analyzed by one-way analysis of variance (ANOVA) with Dunnett's test for multiple post hoc comparisons, and data are expressed as means ± SE (SPSS19.0). Differences were considered statistically significant for *P* < 0.05.

## 3. Results

### 3.1. HPLC-MS Analysis of Components in EFC

**Figure 1:**
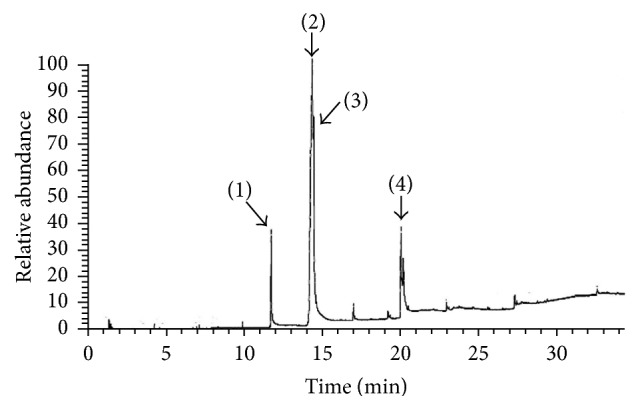
*HPLC chromatogram of EFC*. (1) *n*-Hexadecanoic acid, (2) 9,12-octadecadienoic acid (*ω*6), (3) 9,12,15-octadecatrienoic acid (*ω*3), and (4) butyl 9,12-octadecadienoate.

**Figure 2:**
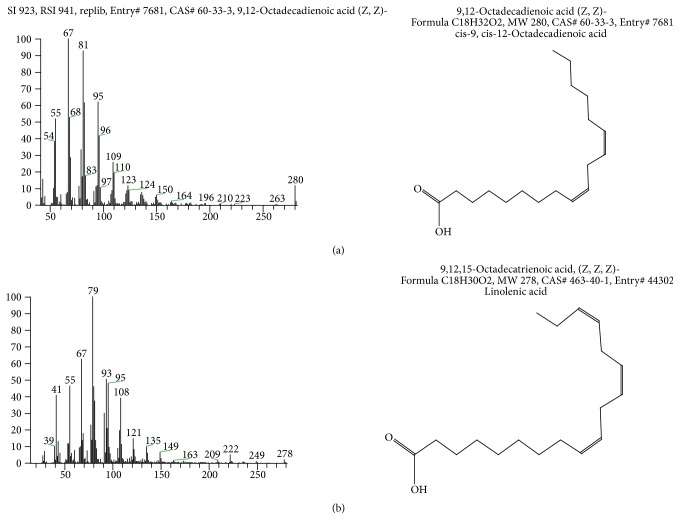
*Graph of mass spectrometry*. (a) 9,12-Octadecadienoic acid (*ω*6) and (b) 9,12,15-octadecatrienoic acid (*ω*3).

It is well known that hempseed is rich in essential fatty acids (EFAs). Therefore, EFC was analyzed by HPLC-MS to identify the components of EFAs. The results show that EFC contains mainly polyunsaturated fatty acids with omega-6 polyunsaturated fatty acids (*ω*-6 PUFAs) and omega-3 polysaturated fatty acids (*ω*-3 PUFAs) in a ratio of 3 : 1. HPLC chromatogram of EFC is shown in Figure 1 and the graph of mass spectrometry is given in Figure 2.

### 3.2. EFC Treatment Improved the General Appearance and Increased the Body Weights and Organ Indices

**Figure 3:**
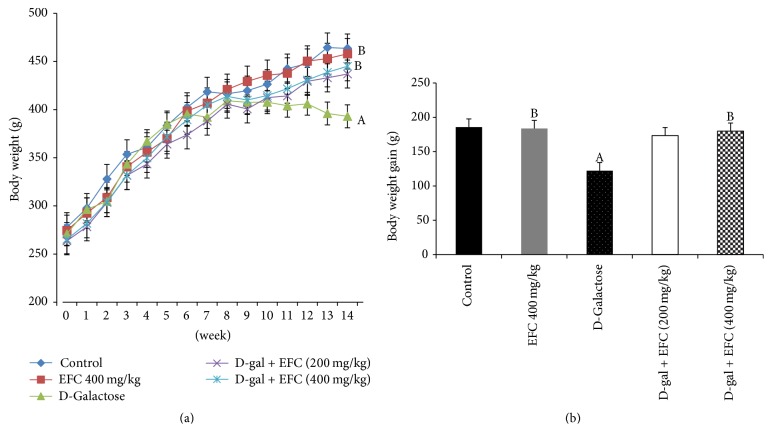
*Effect of EFC on body weight and mean body weight gain in aging rats induced by D-gal*. (a) Effect of EFC on body weight. (b) Mean body weight gain at the end of week 14. The results are presented as mean ± SE (*n* = 8). ^A^*P* < 0.05 as compared to the normal control group. ^B^*P* < 0.05 as compared to the D-gal model group.

**Figure 4:**
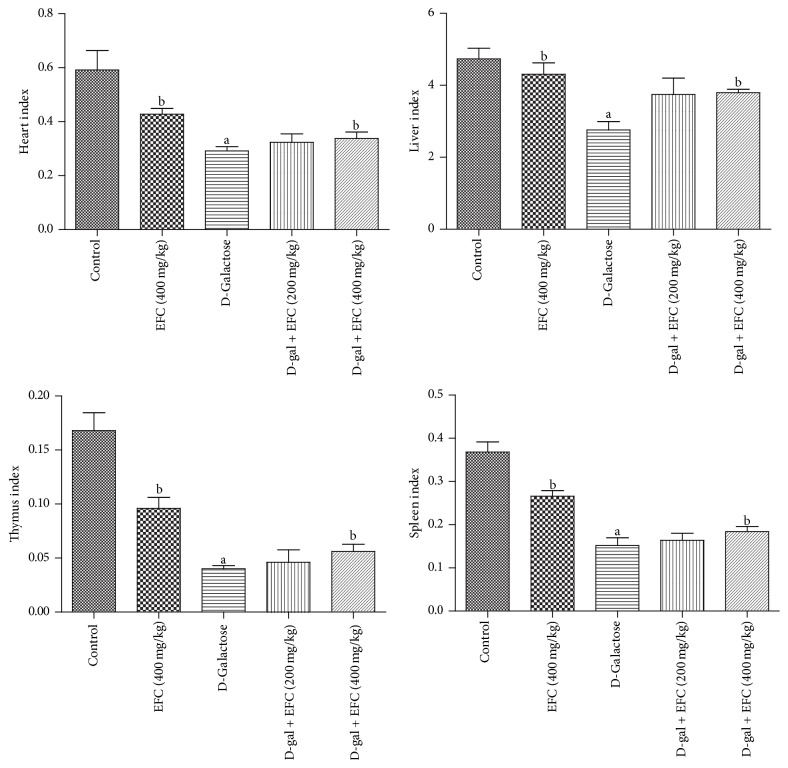
*Effect of EFC on organ indices in aging rats induced by D-gal*. The results are presented as mean ± SE (*n* = 8). ^a^*P* < 0.05 as compared to the control group. ^b^*P* < 0.05 as compared to the D-gal model group.

After 14 weeks of D-gal exposure, rats in the D-gal model group exhibited signs similar to natural aging, such as grayed hair, hair loss, being spiritless, and physical inactivity. Treatment with D-gal plus EFC (400 mg/kg) or EFC treatment (400 mg/kg) improved the general appearance of the rats. The mean body weight in D-gal group notably decreased after 10 weeks, but those of the other groups increased steadily throughout the experiment (Figure 3(a)). Moreover, the mean body weight gain of the D-gal model group was significantly less than that of the control group (*P* < 0.05, Figure 3(b)). However, the increases of body weight in D-gal plus EFC (400 mg/kg) and EFC treatment (400 mg/kg) group were significantly higher than that in the D-gal group (*P* < 0.05). The organ indices, including heart, liver, thymus, and spleen, in the D-gal model group were significantly lower than those of the control group (*P* < 0.05) (Figure 4). However, these parameters turned toward normal after EFC administration.

### 3.3. EFC Protects against the Memory Impairment Caused by D-Gal

**Figure 5:**
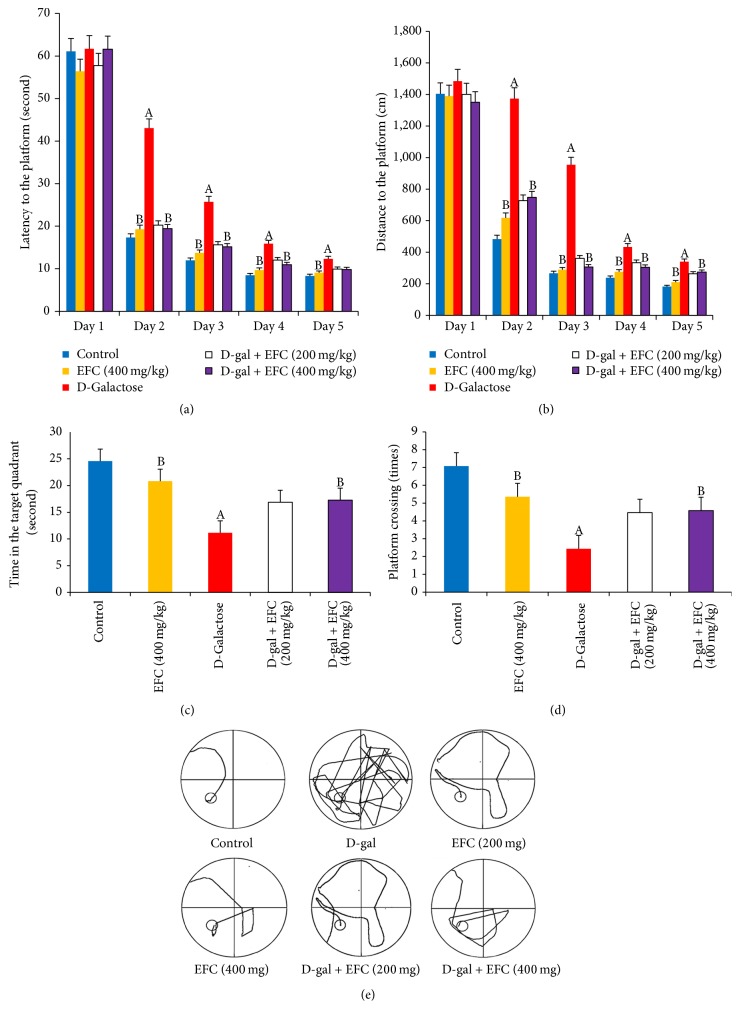
*Effect of EFC on memory impairment induced by D-gal administration (n* = 8* for each group)*. The Morris water maze test was carried out to access the spatial learning and memory ability of rats. (a) Latencies to find a hidden platform in the water maze during the 5 training days. (b) Mean distance to reach the hidden platform during the 5 training days. (c) Time spent in the target quadrant where the platform was located during training days in the probe test. (d) Numbers of crossings of the exact location of the previous platform during the probe test. (e) The representative traces during the probe test. The results are presented as mean ± SE (*n* = 8). ^A^*P* < 0.05 as compared to the normal control group. ^B^*P* < 0.05 as compared to the D-gal model group. Statistical differences between groups were analyzed by one-way analysis of variance (ANOVA) followed by Dunnett's test.

The Morris water maze test is widely used to evaluate the spatial learning and memory. The navigation training showed a decrease in escape latency and distance to target platform in all groups, which demonstrated that rats in all groups have the ability to learn and to navigate to the goal. There was no difference in escape latencies and distances to hidden platform between groups on Day 1 of training. However, from Day 2 to Day 5, the post hoc comparisons revealed that D-gal model group showed longer escape latencies and distances to find the target platform than the control group (*P* < 0.05, Figures 5(a) and 5(b)), whereas rats in D-gal plus EFC (400 mg/kg) and EFC treatment (400 mg/kg) group had shorter latencies and distances to find the hidden platform than the D-gal group (*P* < 0.05).

At the end of the acquisition training, the hidden platform was removed, and a probe trial was used to assess the reference memory. Rats in D-gal plus EFC (400 mg/kg) and EFC treatment (400 mg/kg) group spent more time in the target quadrant than those in D-gal group (*P* < 0.05, Figure 5(c)) and crossed over the location of the previous platform more often than D-gal-treated rats (Figure 5(d)) (*P* < 0.05). These results indicated that EFC treatment prevented the age-related memory deficits induced by D-gal.

Figure 5(e) illustrates representative path-length traces from the last day of navigation training. The rats in D-gal model group took much more time and traveled greater distances to reach the target platform and searched for the goal aimlessly. However, the rats in D-gal plus EFC (400 mg/kg) and EFC treatment (400 mg/kg) group reached the goal in less time and traveled a shorter distance than the D-gal-treated rats.

### 3.4. Effect of EFC on the SOD Activity and MDA Level in Hippocampus Tissue

**Figure 6:**
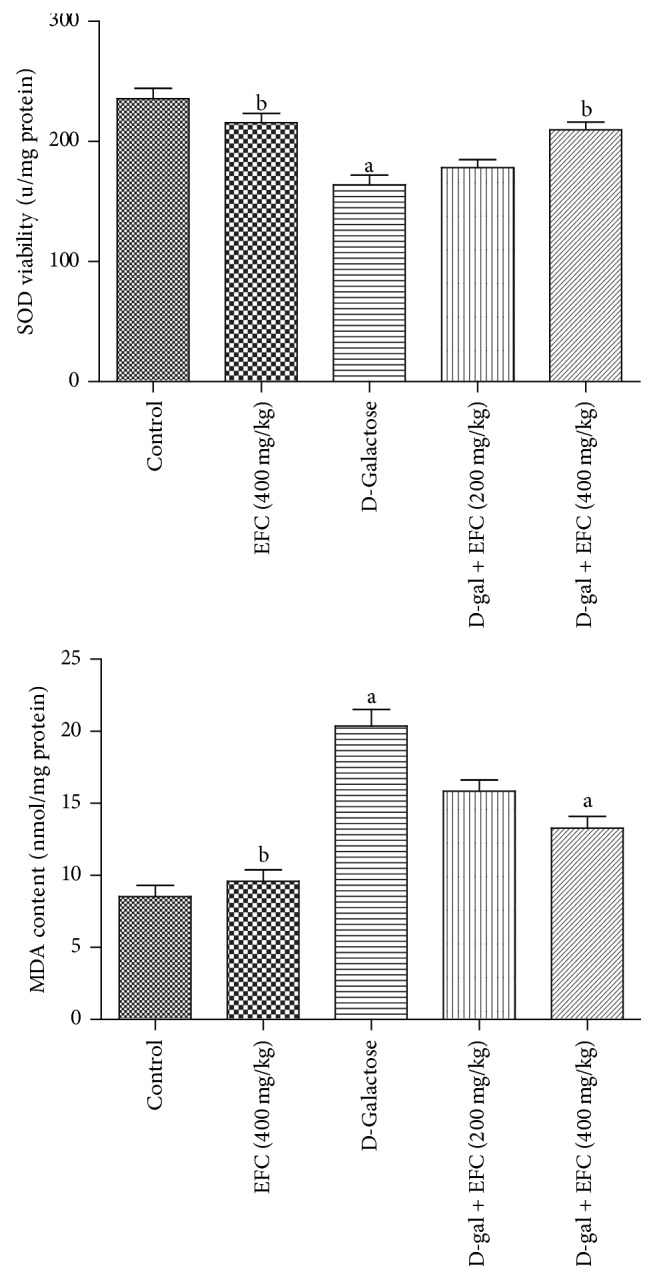
*Effect of EFC on SOD activity and MDA levels in the brain of aging rats induced by D-galactose*. The results are presented as mean ± SE (*n* = 4). ^a^*P* < 0.05 as compared to the control group. ^b^*P* < 0.05 as compared to the D-gal model group.

To assess the effects of EFC on oxidative stress induced by D-gal, we examined SOD activities and MDA levels in the hippocampus. The activities of SOD in the D-gal model group were significantly lower than those of the control group (*P* < 0.05, Figure 6). However, the activities of SOD in D-gal plus EFC (400 mg/kg) and EFC treatment (400 mg/kg) group were significantly higher than that of the D-gal model group (*P* < 0.05). The levels of MDA in the D-gal group in the hippocampus were significantly higher (*P* < 0.05) than those of the normal control group. Additionally, compared with D-gal group with elevated MDA level, the MDA levels of D-gal plus EFC (400 mg/kg) and EFC treatment (400 mg/kg) group notably reduced (*P* < 0.05).

### 3.5. Effect of EFC on the Activation of Astrocytes in Hippocampus Tissue

**Figure 7:**
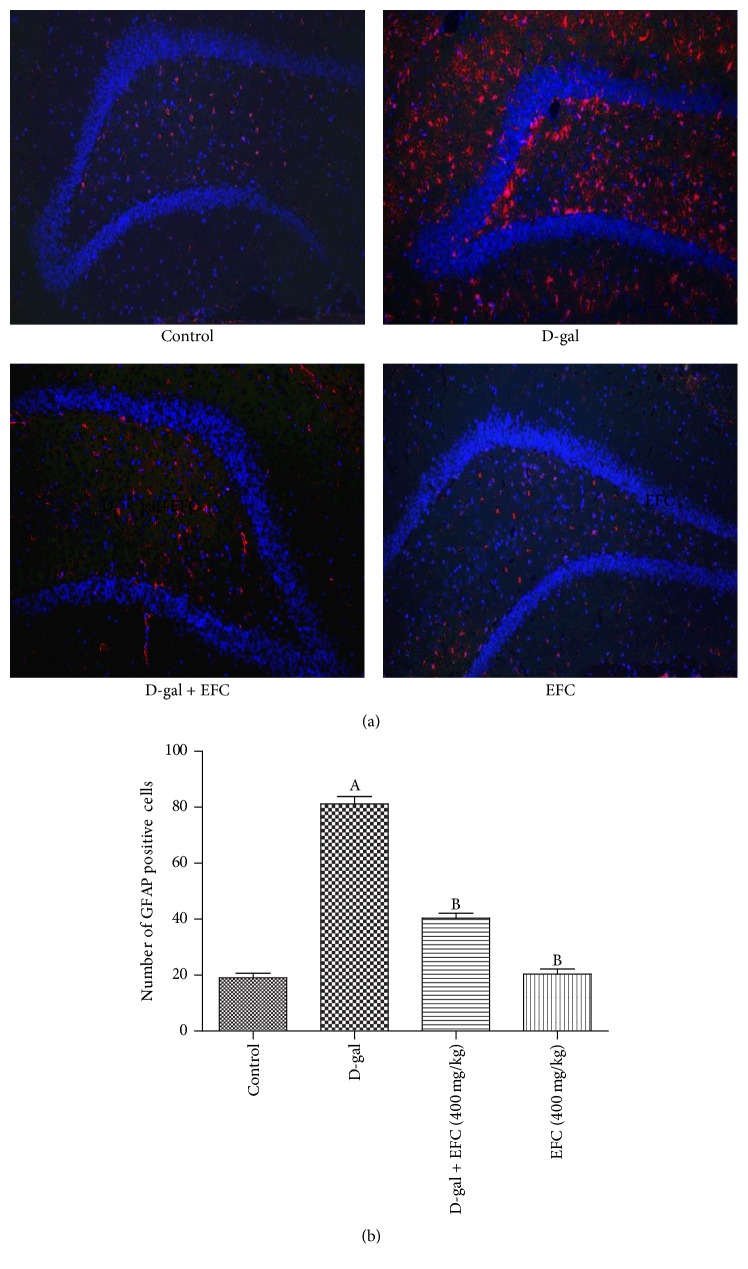
*Effect of EFC on GFAP expression in the dentate gyrus of hippocampus in D-gal-treated rats measured by immunofluorescence (×100)*. (a) The images of immunofluorescence staining of GFAP (red), the scale bar represents 100 *μ*m. (b) The bar graph summarizing the number of GFAP-positive cells. All values are expressed as mean ± SE (*n* = 4), ^A^*P* < 0.05 as compared to the normal control group. ^B^*P* < 0.05 as compared to the D-gal model group.

Astrocytes play critical roles in regulating synaptic transmission and synaptic plasticity which are associated with learning and memory. GFAP is a specific marker for activated astrocytes; thus the expression of GFAP in hippocampal dentate gyrus was examined in the current study. Immunofluorescent analysis showed an increased number of GFAP-stained cells in the dentate gyrus of D-gal treated group compared with control group (*P* < 0.05). However, treatment with EFC significantly reduced the number of GFAP-positive cells in EFC (400 mg/kg) group and D-gal plus EFC (400 mg/kg) group compared with D-gal treated rats (*P* < 0.05, Figure 7).

### 3.6. Effect of EFC on the Expression of the Aging-Related Protein

**Figure 8:**
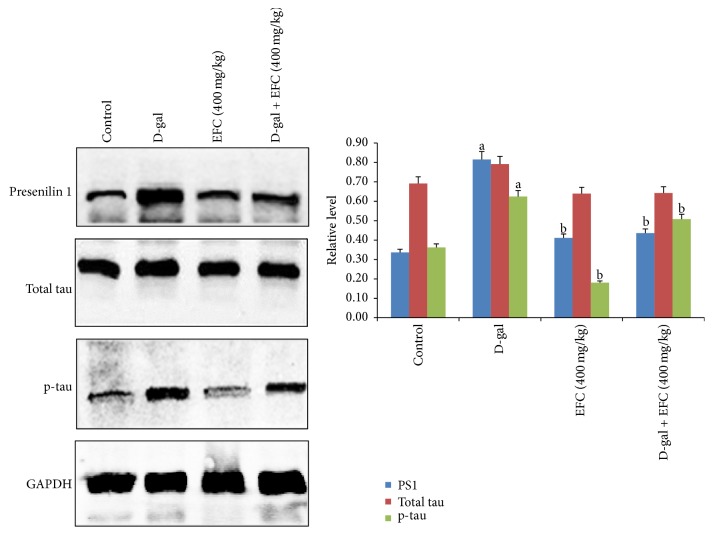
*Effect of EFC on the expression of presenilin 1, total tau, and p-tau in the hippocampus of aging rats induced by D-gal*. The relative protein levels between the tested target protein and internal standard GAPDH were calculated and labeled on *y*-axis. Data are expressed as the mean ± SE (*n* = 4). ^a^*P* < 0.05 as compared to the normal control group. ^b^*P* < 0.05 as compared to the D-gal model group. The bands are from a representative blot. Lane 1: normal control group; lane 2: D-gal-treated group; lane 3: EFC treatment group; lane 4: D-gal plus EFC (400 mg/kg) treatment group.

To investigate whether EFC has potential neuroprotective effect against brain aging, we examined the levels of presenilin 1 (PS1), total tau, and phosphorylated tau. Western blot analysis indicated that the levels of PS1 and the phosphorylation of tau in the hippocampus of the D-gal administration group were significantly higher than those in the control group (*P* < 0.05, Figure 8). However, in D-gal plus EFC (400 mg/kg) and EFC treatment (400 mg/kg) group, expressions of PS1 and phosphorylated tau were significantly lower than those in the D-gal model group (*P* < 0.05), but there was no significant difference in total tau level between groups.

## 4. Discussion

In the current study, we accelerated aging and induced memory impairment by D-galactose in rats model for 14 weeks which mimics the natural aging process in humans [18]. D-gal-injected rats showed increased MDA levels and decreased SOD activities, compared with control group. Additionally, D-gal treatment resulted in significant decrease in organ indices, including heart, liver, thymus, and spleen. Furthermore, notably stimulated astrocytes reactivity indicated as increased GFAP level, as well as hyperphosphorylated tau and overexpression of presenilin 1, were found in the hippocampus of D-gal treated rats. These findings showed that brain oxidative stress, neuroinflammation, and aggregation of neurotoxic proteins were stimulated by D-gal. However, treatment with EFC could reverse these changes and attenuate the effect of aging on learning and memory in D-gal treated rats.

Successively subcutaneous injections of D-gal (100–500 mg/kg) for 8–12 weeks have been widely used to generate a senescence model for oxidative stress and antiaging research [19, 20]. The mechanism of D-gal-induced aging involves an accumulation of galactitol, the abnormal product of D-gal metabolism in cells which causes the generation of ROS [21]. Excessive ROS exposure results in the impairment or loss of enzyme activity and altered cell membrane permeability [22]. Thus, the accumulating galactitol could increase the osmotic stress in cell, which would result in impairment of cellular functions, increased metabolic disorders, and accelerated aging [23, 24]. The present study clearly demonstrates that administration of D-gal at 400 mg/kg per day for 14 weeks caused severe age-related appearance changes and a significant decrease in body weight gain, as well as consequent cognitive impairment associated with aging. However, long-term treatment with EFC partially alleviated these adverse effects.

We investigated the learning and memory ability of rats in a Morris water maze. D-gal-treated rats showed significantly longer escape latencies and distances to the hidden platform and less staying time, as well as more times crossing over the location of the previous platform. However, EFC treatment (400 mg/kg) could attenuate these outcomes. These data are in agreement with previous reports which suggested that the improvement of cognition might be the beneficial effect of polyunsaturated fatty acids enriched in the hempseeds [25, 26].

We found that SOD activity was significantly decreased in D-gal treated rats, and MDA content was dramatically increased. However, EFC reversed these changes in the D-gal-treated rats by enhancing the antioxidization capacity. Senescence is the result of oxidative stress, according to the free radical theory of aging [27]. D-gal induces oxidative stress and generates ROS. D-gal overload may induce changes that mimic the normal aging process in rodents [28–30], and oxidative stress caused by D-gal has an important role in the age-associated cognitive decline [5, 31]. There is a growing consensus that SOD is an important antioxidant enzyme that acts as the first line of defense [32], and increased MDA is an important marker for lipid peroxidation, which is an indicator of oxidative damage of membranes. Hence, increasing SOD viability and decreasing levels of MDA are beneficial for preventing oxidative damage [33].

After 14 weeks of D-gal injection, the heart and liver indices of rats were notably decreased, which implying that aging was accelerated. To the best of our knowledge, the heart and the liver are both important metabolic organs and the function of heart is to provide the pumping pressure that allows blood flow deliver adequate oxygen and nutrients to organs and tissues. The liver is the main part of biological oxidation, participating in glucose, lipid, and protein metabolism as well as immune defense. Meanwhile, the thymus and the spleen indices of rats were significantly decreased, indicating that D-gal can also cause the atrophy of immune organs, as well as the declined immune function, which may be one of the reasons of immune aging. These results support the concept that thymus is the first organ to be sensitive to aging in the body, and thymus atrophy is the most obvious immunologic change in aging [34]. Thus, the thymus and spleen indices could be used to evaluate aging [35]. Herein, as in D-gal-treated rats, EFC significantly increased the function of heart and liver as well as the immune system as Luo and colleagues observed in amnesic mice [36].

Astrocytes are the main components of glial cells in the nervous system, mounting evidence indicated that neuroinflammation is a result of activation of astrocytes which leads to the memory impairment in the D-gal aging mouse model [5]. Our result in the present study clearly demonstrated that 14 weeks of D-gal administration significantly stimulated astrocytes reactivity indicated as increased GFAP level. However, EFC treatment could reverse these changes, indicating that activation of astrocytes may trigger memory impairment and the reversal of activation of astrocytes by EFC treatment might be an outcome of the anti-neuroinflammation. Therefore, EFC improved the cognitive deficits induced by D-gal administration partly through the inhibition of inflammatory responses in brain.

To further investigate the possible mechanism of EFC in ameliorating the memory decline in D-gal administration rats, we examined the levels of total tau, phosphorylated tau, and PS1. Tau is a microtubule-associated protein; its main functions are to facilitate the assembly and maintenance of microtubules and to maintain axonal transport in neurons [37]. To the best of our knowledge, senile plaques and neurofibrillary tangle (NFT) are two main pathological features of AD and hyperphosphorylated tau is the main component of NFT [38, 39]. When tau is hyperphosphorylated and aggregates in the neuron, it becomes defective and no longer stabilizes microtubules properly [40]. Moreover, hyperphosphorylated tau forms paired helical filaments (PHF) that weaken its binding to microtubules [41]. The death of neurons and cognitive dysfunction are related to the excessive phosphorylation of tau protein. Furthermore, hyperphosphorylated tau is the main component of NFTs, and NFT formation is considered to be an early pathological change in AD [42]. In addition, the number of NFTs in the CA1 of hippocampus seems to be closely related to cognitive status [43]. By western blot analysis, we found tau phosphorylation at Ser396 in D-gal model group was significantly higher than that in the control group. However, EFC (400 mg/kg) significantly attenuated tau phosphorylation. These results showed EFC could reduce the phosphorylation level of tau to ameliorate memory impairment induced by D-gal.

The present study demonstrated that PS1 expression was markedly higher in the aging model than the control group, and that EFC (400 mg/kg) significantly decreased PS1 expression. The results indicated that treatment with EFC could improve spatial learning and memory in D-gal-treated rats by attenuating tau phosphorylation and by suppressing expression of PS1, which are related to brain aging. Mutations in PS1 are the major cause of the most aggressive form of familial Alzheimer's disease (FAD) which leads to defective learning and impaired memory [44, 45]. In addition, PS1 represents the catalytic components of protease complexes that directly cleave the amyloid precursor protein (APP) and is responsible for most amyloid *β*-peptide (A*β*) production. Alzheimer-associated mutations in PS1 increase production of A*β*42 (the neurotoxic form of A*β*), resulting in deposition of A*β* and senile plaques which is a hallmark of AD [46]. Furthermore, PS1 mutant knockin mice have spatial memory deficits in the Morris water maze and PS1 mutations show age-dependent effects on synaptic plasticity and learning and memory [47]. On the other hand, the overexpression of PS1 can also affect the process of tau phosphorylation. The underlying mechanism lies in the interaction of tau and glycogen synthase kinase 3 beta (GSK-3*β*), PS1 could directly bind tau and GSK-3*β* to the same region of PS1, which regulates phosphorylation of tau [48]. Moreover, PS1 overexpression inhibited neurite growth and promoted neuritic dystrophy and tangle formation by interfering with Notch 1 signaling and enhancing pathological changes in tau [49].

## 5. Conclusion

**Figure 9:**
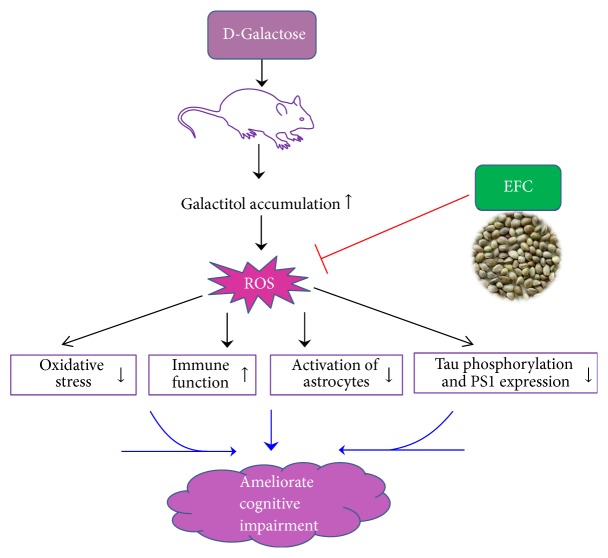
*Proposed mechanism of the effect of EFC on ameliorating cognitive impairment induced by D-gal*. EFC could relieve age-related cognitive impairment by attenuating oxidative stress, improving immune function, and inhibiting the activation of astrocytes as well as attenuating tau phosphorylation and suppressing PS1 expression in the brain of D-gal treated rats.

In summary, we found that EFC mitigated the features of aging induced by D-gal in rats and improved behavioral performance in Morris water maze. The antiaging effects of EFC include reducing oxidative stress, improving immune function, and inhibiting the activation of astrocytes as well as attenuating tau phosphorylation and suppressing PS1 expression (Figure 9). Therefore, our findings suggest that EFC would be useful for relieving age-related memory impairments.

## Abbreviations

EFC: Extract of Fructus Cannabis
D-gal: D-Galactose
AD: Alzheimer's disease
HPLC-MS: High performance liquid chromatography-mass spectrometry
MWM: Morris water maze
SOD: Superoxide dismutase
MDA: Malondialdehyde
PUFA: Polyunsaturated fatty acid
EFAs: Essential fatty acids
BDNF: Brain-derived neurotrophic factor
GFAP: Glial fibrillary acidic protein
PS1: Presenilin-1
NFT: Neurofibrillary tangle.

## References

[1] Kanasi E., Ayilavarapu S., Jones J. (2016). The aging population: demographics and the biology of aging. *Periodontology 2000*.

[2] Harman D. (2009). Origin and evolution of the free radical theory of aging: a brief personal history, 1954–2009. *Biogerontology*.

[3] Lü J.-M., Lin P. H., Yao Q., Chen C. (2010). Chemical and molecular mechanisms of antioxidants: experimental approaches and model systems. *Journal of Cellular and Molecular Medicine*.

[4] Qu Z., Yang H., Zhang J. (2016). Cerebralcare granule*Ⓡ*, a chinese herb compound preparation, attenuates D-galactose induced memory impairment in mice. *Neurochemical Research*.

[5] Ali T., Badshah H., Kim T. H., Kim M. O. (2015). Melatonin attenuates D-galactose-induced memory impairment, neuroinflammation and neurodegeneration via RAGE/NF-KB/JNK signaling pathway in aging mouse model. *Journal of Pineal Research*.

[6] Lu J., Wu D.-M., Zheng Y.-L. (2010). Ursolic acid attenuates D-galactose-induced inflammatory response in mouse prefrontal cortex through inhibiting AGEs/RAGE/NF-*κ*B pathway activation. *Cerebral Cortex*.

[7] Wei H., Li L., Song Q., Ai H., Chu J., Li W. (2005). Behavioural study of the D-galactose induced aging model in C57BL/6J mice. *Behavioural Brain Research*.

[8] Gao J., He H., Jiang W. (2015). Salidroside ameliorates cognitive impairment in a d-galactose-induced rat model of Alzheimer's disease. *Behavioural Brain Research*.

[9] Lee M. J., Park S. H., Han J. H. (2011). The effects of hempseed meal intake and linoleic acid on drosophila models of neurodegenerative diseases and hypercholesterolemia. *Molecules and Cells*.

[10] Callaway J. C. (2004). Hempseed as a nutritional resource: an overview. *Euphytica*.

[11] Girgih A. T., Alashi A. M., He R. (2014). A novel hemp seed meal protein hydrolysate reduces oxidative stress factors in spontaneously hypertensive rats. *Nutrients*.

[12] Richard M. N., Ganguly R., Steigerwald S. N., Al-Khalifa A., Pierce G. N. (2007). Dietary hempseed reduces platelet aggregation. *Journal of Thrombosis and Haemostasis*.

[13] Girgih A. T., Alashi A., He R., Malomo S., Aluko R. E. (2014). Preventive and treatment effects of a hemp seed (Cannabis sativa L.) meal protein hydrolysate against high blood pressure in spontaneously hypertensive rats. *European Journal of Nutrition*.

[14] Peng J.-H., Liu C.-W., Pan S.-L. (2017). Potential unfavorable impacts of BDNF Val66Met polymorphisms on metabolic risks in average population in a longevous area. *BMC Geriatrics*.

[15] Luo J., Yin J.-H., Wu H.-Z., Wei Q. (2003). Extract from Fructus cannabis activating calcineurin improved learning and memory in mice with chemical drug-induced dysmnesia. *Acta Pharmacologica Sinica*.

[16] Li J.-J., Zhu Q., Lu Y.-P. (2015). Ligustilide prevents cognitive impairment and attenuates neurotoxicity in d-galactose induced aging mice brain. *Brain Research*.

[17] Zhang Y., Lv X., Bai Y. (2015). Involvement of sigma-1 receptor in astrocyte activation induced by methamphetamine via up-regulation of its own expression. *Journal of Neuroinflammation*.

[18] Yoo D. Y., Kim W., Lee C. H. (2012). Melatonin improves D-galactose-induced aging effects on behavior, neurogenesis, and lipid peroxidation in the mouse dentate gyrus via increasing pCREB expression. *Journal of Pineal Research*.

[19] Ruan Q., Hu X., Ao H. (2014). The neurovascular protective effects of huperzine A on D-galactose-induced inflammatory damage in the rat hippocampus. *Gerontology*.

[20] Xian Y.-F., Su Z.-R., Chen J.-N. (2014). Isorhynchophylline improves learning and memory impairments induced by D-galactose in mice. *Neurochemistry International*.

[21] Çoban J., Doğan-Ekici I., Aydın A. F., Betül-Kalaz E., Doğru-Abbasoğlu S., Uysal M. (2015). Blueberry treatment decreased D-galactose-induced oxidative stress and brain damage in rats. *Metabolic Brain Disease*.

[22] Kumar H., Lim H. W., More S. V. (2012). The role of free radicals in the aging brain and Parkinson's disease: convergence and parallelism. *International Journal of Molecular Sciences*.

[23] Zhang C. E., Wei W., Liu Y. H. (2012). Establishment and evaluation of subacute aging rat model induced by D-galactose. *Chinese Journal of Gerontology*.

[24] Lin Z. M., Chen L. M., L Z. M., Xia X. (2016). The effect of different extracts from Hemp seed in mice with experimental Alzheimers disease. *Pharmacology and Clinics of Chinese Materia*.

[25] Chen T., He J., Zhang J. (2010). Analytical characterization of hempseed (Seed of Cannabis sativa L.) oil from eight regions in China. *Journal of Dietary Supplements*.

[26] Su H.-M. (2010). Mechanisms of n-3 fatty acid-mediated development and maintenance of learning memory performance. *Journal of Nutritional Biochemistry*.

[27] Oliveira B. F., Nogueira-Machado J. A., Chaves M. M. (2010). The role of oxidative stress in the aging process. *TheScientificWorldJournal*.

[28] Cui X., Zuo P., Zhang Q. (2006). Chronic systemic D-galactose exposure induces memory loss, neurodegeneration, and oxidative damage in mice: protective effects of R-alpha-lipoic acid. *Journal of Neuroscience Research*.

[29] Lei M., Su Y., Hua X. (2008). Chronic systemic injection of D-galactose impairs the septohippocampal cholinergic system in rats. *NeuroReport*.

[30] Dulcy C. P., Singh H. K., Preethi J., Rajan K. E. (2012). Standardized extract of *Bacopa monniera* (BESEB CDRI-08) attenuates contextual associative learning deficits in the aging rat's brain induced by D-galactose. *Journal of Neuroscience Research*.

[31] Kumar A., Prakash A., Dogra S. (2011). Centella asiatica attenuates d-galactose-induced cognitive impairment, oxidative and mitochondrial dysfunction in mice. *International Journal of Alzheimer's Disease*.

[32] Rizvi S. I., Maurya P. K. (2007). Alterations in antioxidant enzymes during aging in humans. *Molecular Biotechnology*.

[33] Tian Y., Zou B., Yang L. (2011). High molecular weight persimmon tannin ameliorates cognition deficits and attenuates oxidative damage in senescent mice induced by D-galactose. *Food and Chemical Toxicology*.

[34] Feng R., He W., Ochi H. (2001). A new murine oxidative stress model associated with senescence. *Mechanisms of Ageing and Development*.

[35] Doan V. M., Chen C., Lin X., Nquyen V. P., Li W., Chen Q. (2015). Yulangsan polysaccharide improves redox homeostasis and immune impairment in D-galactose-induced mimetic aging. *Food & Function*.

[36] Luo J., Yin J.-H., Wei Q. (2003). The effect of calcineurin activator, extracted from Chinese herbal medicine, on memory and immunity in mice. *Pharmacology Biochemistry and Behavior*.

[37] Avila J., Lucas J. J., Pérez M., Hernández F. (2004). Role of tau protein in both physiological and pathological conditions. *Physiological Reviews*.

[38] Ballatore C., Lee V. M.-Y., Trojanowski J. Q. (2007). Tau-mediated neurodegeneration in Alzheimer's disease and related disorders. *Nature Reviews Neuroscience*.

[39] Chun W., Johnson G. V. (2007). The role of tau phosphorylation and cleavage in neuronal cell death. *Frontiers in Bioscience*.

[40] Kim D. H., Yeo S. H., Park J.-M. (2014). Genetic markers for diagnosis and pathogenesis of Alzheimer's disease. *Gene*.

[41] Maas T., Eidenmüller J., Brandt R. (2000). Interaction of tau with the neural membrane cortex is regulated by phosphorylation at sites that are modified in paired helical filaments. *The Journal of Biological Chemistry*.

[42] Grundke-Iqbal I., Iqbal K., Quinlan M., Tung Y. C., Zaidi M. S., Wisniewski H. M. (1986). Microtubule associated protein tau. A component of Alzheimer paired helical filaments. *The Journal of Biological Chemistry*.

[43] Xekardaki A., Kövari E., Gold G. (2015). Neuropathological changes in aging brain. *Advances in Experimental Medicine and Biology*.

[44] Wang R., Dineley K. T., Sweatt J. D., Zheng H. (2004). Presenilin 1 familial Alzheimer's disease mutation leads to defective associative learning and impaired adult neurogenesis. *Neuroscience*.

[45] Sun X., Beglopoulos V., Mattson M. P., Shen J. (2005). Hippocampal spatial memory impairments caused by the familial Alzheimer's disease-linked presenilin 1 M146V mutation. *Neurodegenerative Diseases*.

[46] Wolfe M. S., Yankner B. A. (2016). Sorting Out Presenilins in Alzheimer's Disease. *Cell*.

[47] Auffret A., Gautheron V., Mattson M. P., Mariani J., Rovira C. (2010). Progressive age-related impairment of the late long-term potentiation in Alzheimer's disease presenilin-1 mutant knock-in mice. *Journal of Alzheimer's Disease*.

[48] Takashima A., Murayama M., Murayama O. (1998). Presenilin 1 associates with glycogen synthase kinase-3*β* and its substrate tau. *Proceedings of the National Academy of Sciences of the United States of America*.

[49] Pigino G., Pelsman A., Mori H., Busciglio J. (2001). Presenilin-1 mutations reduce cytoskeletal association, deregulate neurite growth, and potentiate neuronal dystrophy and tau phosphorylation. *Journal of Neuroscience*.

